# DNA-mediated adjuvant immunotherapy extends survival in two different mouse models of myeloid malignancies

**DOI:** 10.18632/oncotarget.5572

**Published:** 2015-09-10

**Authors:** Carole Le Pogam, Satyananda Patel, Petra Gorombei, Laura Guerenne, Patricia Krief, Nader Omidvar, Nilgun Tekin, Elena Bernasconi, Flore Sicre, Marie-Helene Schlageter, Martine Chopin, Maria-Elena Noguera, Robert West, Ansu Abu, Vikram Mathews, Marika Pla, Pierre Fenaux, Christine Chomienne, Rose Ann Padua

**Affiliations:** ^1^ Unité Mixte de la Recherche de Santé (UMR-S), Institut Universitaire d'Hématologie, Université Paris Diderot, Paris, France; ^2^ Institut National de la Santé et de la Recherche Médicale (INSERM) Unité (U), Paris, France; ^3^ Hôpital Saint Louis, Assistance Publique-Hôpitaux de Paris (AP-HP), Paris, France; ^4^ Haemotology Department, Cardiff University School of Medicine, Cardiff, UK; ^5^ Biotechnology Institute, Ankara University, Ankara, Turkey; ^6^ Département d'Expérimentation Animale, Institut Universitaire d'Hématologie, University Paris Diderot, Paris, France; ^7^ Welsh Heart Research Institute, Cardiff University School of Medicine, Cardiff, UK; ^8^ Department of Hematology, Christian Medical College and Hospital, Vellore, India

**Keywords:** plasmid DNA based immunotherapy, MDS, APL, memory T-cells, Immunology and Microbiology Section, Immune response, Immunity

## Abstract

We have previously shown that a specific promyelocytic leukemia-retinoic acid receptor alpha (PML-RARA) DNA vaccine combined with *all-trans* retinoic acid (ATRA) increases the number of long term survivors with enhanced immune responses in a mouse model of acute promyelocytic leukemia (APL). This study reports the efficacy of a non-specific DNA vaccine, pVAX14Flipper (pVAX14), in both APL and high risk myelodysplastic syndrome (HR-MDS) models. PVAX14 is comprised of novel immunogenic DNA sequences inserted into the pVAX1 therapeutic plasmid. APL mice treated with pVAX14 combined with ATRA had increased survival comparable to that obtained with a specific PML-RARA vaccine. Moreover, the survival advantage correlated with decreased PML-RARA transcript levels and increase in anti-RARA antibody production. In HR-MDS mice, pVAX14 significantly improved survival and reduced biomarkers of leukemic transformation such as phosphorylated mitogen-activated protein/extracellular signal-regulated kinase kinase (MEK) 1. In both preclinical models, pVAX14 vaccine significantly increased interferon gamma (IFNγ) production, memory T-cells (memT), reduced the number of colony forming units (CFU) and increased expression of the adapter molecule signalling to NF-κB, *MyD88*. These results demonstrate the adjuvant properties of pVAX14 providing thus new approaches to improve clinical outcome in two different models of myeloid malignancies, which may have potential for a broader applicability in other cancers.

## INTRODUCTION

A DNA vaccine is composed of a plasmid DNA that encodes an antigen of interest or an immunogenic sequence [1, 2, 3] Although DNA-based strategies have emerged as a promising approach to immunotherapy development, they suffer from low immunogenicity which limits their effectiveness; hence emphasis is now on the importance of adjuvants as crucial components of successful vaccines. Furthermore, recent studies have been focused on strategies to improve the immunogenicity of DNA vaccines [4, 5].

Targeted therapies for hematological malignancies have matured since the advent of *all-trans* retinoic acid (ATRA) to treat acute promyelocytic leukemia (APL) [6]. APL is a specific subtype of acute myeloid leukemia (AML) characterized by the t(15;17) translocation resulting in a PML-RARA (for promyelocytic leukemia-retinoic acid receptor alpha) fusion protein. Boosting the immune system of leukemia patients in complete remission offers a novel approach. In previous studies, we demonstrated that a specific *PML-RARA* DNA vaccine, when combined with ATRA, improved survival over treatment with ATRA alone, with a protective effect that was B and T-cell mediated [7, 8, 9].

However, most hematological malignancies lack specific oncoproteins, making specific DNA immunotherapies inappropriate. This is particularly the case for myelodysplastic syndromes (MDS), characterized by ineffective hematopoiesis leading to blood cytopenias, frequent progression to AML, and which generally remain, despite recent therapeutic progress with azacitidine (AZA), incurable. Allogeneic stem cell transplant (SCT), whose efficacy largely relies on immunotherapy, remains the only curative treatment of MDS [10]. Nevertheless, only very few high risk (HR)-MDS patients may benefit from allogeneic SCT due to the median age and the need of a human leukocyte antigen (HLA) compatible donor. Immunotherapeutic approaches in HR-MDS can take advantage of the immune surveillance elicited by the MDS malignant clone in MDS patients. Indeed, MDS, before it transforms into AML, is characterized by an increased apoptosis of hematopoietic precursors, potentially resulting in tumor antigens being presented to the immune system and evoking an adaptive immune response. Activated T cells and clonal T-cell expansions are found in some MDS patients [11, 12] with reported decreased frequencies of regulatory T-cells, further documenting the role of immune surveillance [11].

In order to develop a DNA adjuvant approach to enhance endogenous anti-tumor immune response we designed a pVAX1-based DNA vaccine. In this study, we characterized a non-specific vaccine, designated pVAX14Flipper (pVAX14) and we evaluated its potential therapeutic effects in two different preclinical models of myeloid malignancies.

## RESULTS

### Impact of the pVAX14 vaccine on survival and tumor burden of APL and HR-MDS mice

**Figure 1:**
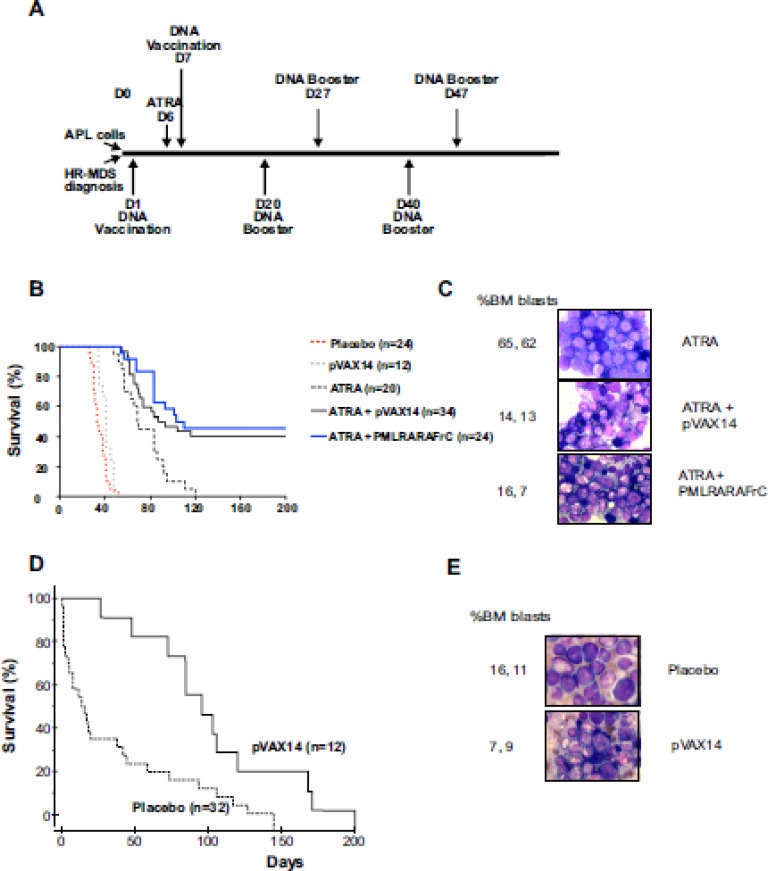
Increased survival of APL and HR-MDS mice by pVAX14 **A.** Schematic diagram of the protocols used. For APL (upper part) spleen blast cells were injected IV (D0), followed by ATRA (5mg-21-day release pellet) on day 6 (D6) + placebo (HBSS) or DNA (2×50 μg) was administered IM on day 7 (D7) and every 20 days for a total of 3 cycles. The protocol for HR-MDS (lower part) is illustrated. At diagnosis (platelet below 1300 K/μl) the mice were treated with either placebo (HBSS) or pVAX14 DNA every 20 days for a total of 3 injections (IM 2×50ug); **B.** Kaplan-Meier survival curves showing increased survival in the APL mice; ATRA+pVAX14 and the specific vaccine ATRA+ *PML-RARA* showed the best survival with no significant difference between the two treatments. The statistical table for all comparisons of this figure is shown in Supplementary Table S1; **C.** Giemsa stained BM of APL mice at day 160 treated with ATRA, ATRA+pVAX14 or ATRA+*PML-RAR*, percentage BM blasts were determined after counting 200 cells; **D.** Kaplan-Meier survival curves showing extended life-span of HR-MDS mice treated with pVAX14; mice treated with pVAX14 (solid line) compared with untreated/vehicle treated controls (dashed line) plotted from date of diagnosis extends life-span of HR-MDS mice (*p* < 0.0001); e) Giemsa stained BM of HR-MDS mice at day 40 treated with placebo (HBSS) and pVAX14, percentage BM blasts were determined after counting 200 cells; the Mantel-Cox (log-rank) test was used to compare the percent survival of different groups. The Prism software was used for the Mantel-Cox (log-rank) test analysis

**Figure 2:**
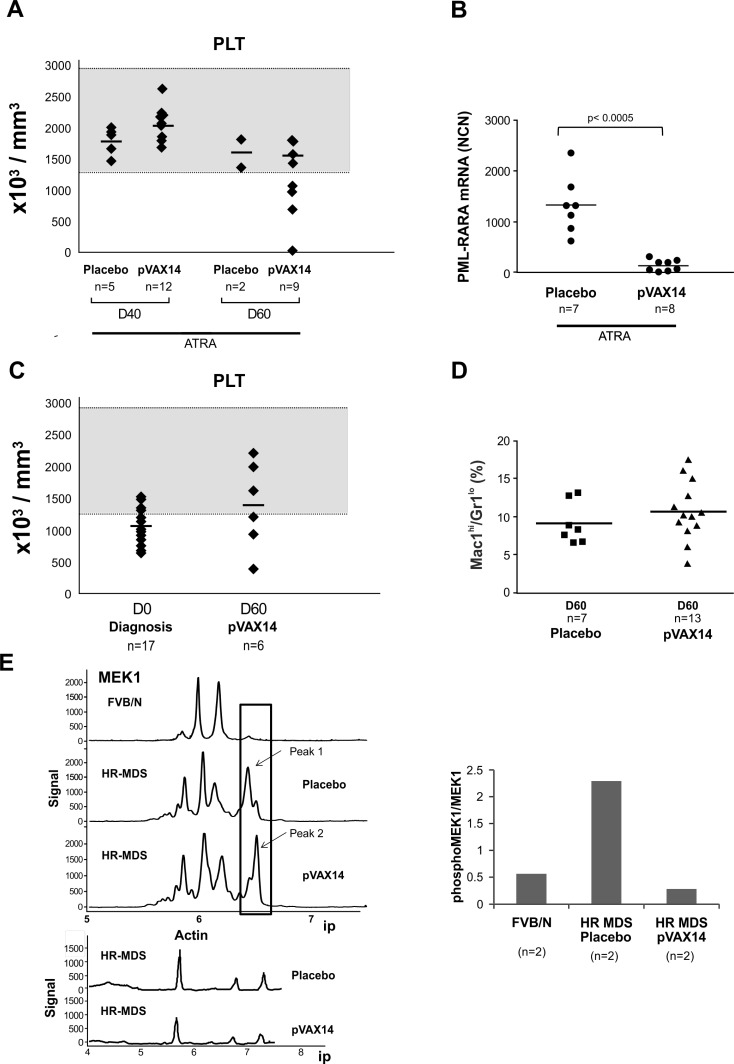
Effect of pVAX14 on disease **A.** PB platelet counts (PLT) of ATRA or ATRA+pVAX14 treated mice; on days (D) 40 and 60; by D60 only 2 mice in the ATRA treated group remain; **B.** minimal residual disease (MRD) in APL mice treated with ATRA and pVAX14; a significant reduction in MRD was observed on day 50 of ATRA (5mg) + pVAX14-treated APL mice; mice were treated with ATRA or in combination with pVAX14. Results were expressed as normalized copy numbers (NCN) of *PML-RARA* transcripts using *Abl* as a housekeeping gene. The difference between these 2 groups were significant (*p* < 0.0005); **C.** PB platelet counts (PLT) of HR-MDS mice treated with placebo (HBSS), at diagnosis (D0) and after treatment with pVAX14 at day 60 (D60). Mice with platelet counts lower or just above 1300K/μl were considered as diseased; the normal range is shaded grey. There were no statistical differences between pre and post pVAX14 treatment on Day 60, although 3/6 treated mice were in the normal range; **D.** persistence of circulating PB blasts indicative of disease in the MDS mice with pVAX14 compared to placebo (HBSS) as seen in the Mac-1^+^/Gr-1^lo^ population, which remained stable during the 60 days of treatment and follow up; **E.** Modifications of MEK1 in spleen cells of HR-MDS and normal FVB/N mice on day 30. Representative NanoPro traces showing MEK1 spleen extracts from HR-MDS treated with either placebo (HBSS) or pVAX14. The isoelectric points (ip) are shown in the X axis. Boxed are Peak 1 representing a phosphorylated isoform (arrowed) and peak 2, a dephosphorylated isoform (arrowed). Quantitations are shown in histogram (*n* = 2 in triplicate) expressed as a ratio of pMEK1/MEK1. Actin was used as a housekeeping protein to control for proteins loading of mice treated with placebo and or with pVAX14. Nonparametric, unpaired, two tailed, Mann-Whitney test was used to compare different groups. The Prism software was used for the Mann-Whitney test analysis.

To assess the efficacy of the pVAX14 vaccine, we first used, as a proof of concept, the APL mouse model [13] where we previously identified and documented the enhanced survival induced by the specific vaccine *PML-RARA* in combination with ATRA compared with either ATRA alone or ATRA+pcDNA_3_ empty vector [8]. In the APL preclinical model (protocol in Figure 1A, upper part) treatment with ATRA alone controlled disease up to 50 days. Survival of the APL mice treated by ATRA in combination with the specific *PML-RARA* as well as with the non-specific pVAX14 was significantly (*p* < 0.0001 and *p* < 0.0014, respectively) superior to that of the mice treated by ATRA alone (Figure 1B, Supplementary Table S1). As we previously reported with the specific vaccine [7] a significant reduction of bone marrow (BM) APL blasts and increase in presence of differentiated cells was also observed in ATRA+pVAX14-treated mice on day 160 (Figure 1C). After day 50 relapses were seen with the recurrence of thrombocytopenia (Figure 2A). As in our *PML-RARA* published study [14], the low levels of *PML-RARA* transcripts of the ATRA+pVAX14-treated mice (Figure 2B) correlated with the achieved survival advantage and with rescue of thrombocytopenia in some of the mice (Figure 2A). The combined results of enhanced survival, MRD, platelet monitoring and the clearance of BM APL blasts demonstrated that in this APL preclinical model the non-specific pVAX 14 had an anti-leukemic effect.

Using a similar protocol in the HR-MDS preclinical model [15] (Figure 1A, lower part), treatment with pVAX14 significantly extended survival compared to placebo-treated mice (Figure 1D, *p* < 0.0001) with a reduction of BM blast cells by day 40 (Figure 1E). As shown in Figure 2C and 2D, some of the pVAX14-treated MDS mice had a recovery of platelet counts, which correlated with increased survival and no significant changes in circulating blasts (Mac-1^+^/Gr-1^lo^). We have been looking for objective parameters of controlled MDS disease and we took advantage of our data showing disease-associated changes in one of the MEK isoforms. As shown in Figure 2E, when MEK1 profiles were analysed using the Nanopro assay, we have been able to identify the MEK1 isoform (peak 1 arrowed) present only in HR-MDS mice. This MEK1 isoform showed qualitative changes (dephosphorylation) when spleen samples from HR-MDS mice treated with pVAX14 were analysed (Figure 2E - peak 2 arrowed). Thus our data showed that treatment with a non-specific pVAX14 vaccine can have a pronounced effect on the survival of HR-MDS mice.

### Characteristics of the pVAX14 vaccine construct

**Figure 3:**
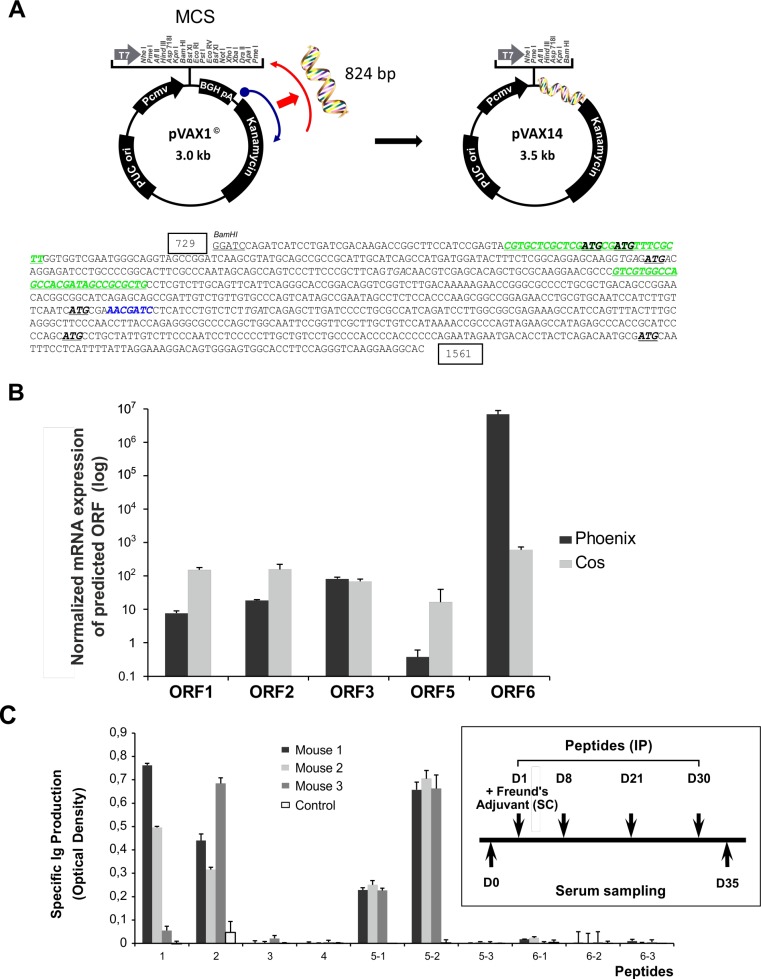
Cloning and characterization of pVAX14 **A.** Schematic diagram showing the cloning of the 824 bp insert into the BamHI site of the multiple cloning site (MCS) of the pVAX1 plasmid vector, generating the pVAX14 clone, the sequence of the insert comprising of the kanamycin resistance gene showing the GC-rich regions in green italics, the single consensus human specific CpG is in blue. The kanamycin resistance region is rotated 180° and the antisense reversed strand is coding, effectively flipped, hence the name Flipper. The 6 Open reading frames (ORFs) are depicted with start codons ATG in bold and stop codons TGA are in italics. The nucleotide numbers are boxed to coincide with the numbers on Supplemental Figure S1A; **B.** Expression of mRNA transcripts from the predicted ORFs. Expression was performed in COS and Phoenix cells. Gene expression was calculated for each probe set using the ΔΔCt method of empty vector (pVAX1) or pVAX14 against the untransfected RNA, all normalized against the ABL gene. Normalized pVAX1 was subtracted from normalized pVAX14 and plotted on a log scale. The ORF 4 is too small (29bp) to assay. Data are represented as mean +/− SEM of triplicate reactions; **C.** Identification of immunogenic peptides predicted from the inserted sequence (Supplementary Table S3). Four synthetic peptides are immunogenic in FVB/N mice as measured by the Ig production (Optical density); Open reading frames (ORFs) 1, 2 and 5 are immunogenic; inset shows protocol for induction of immune responses with the synthetic peptides. FVB/N mice (mouse no.1-3), *n* = 3 for each peptide (100 μg) were injected subcutaneously (SC) with Complement Freund's Adjuvant (CFA) on day 1 and the peptides were injected intraperitoneally (IP) (D1, D8, D21 and D30). Sera were collected before immunization and at day 35. Ig production was measured by measuring the optical density at a wavelength of 495 nm. Control mice were injected with PBS.

Analysis of the sequence of the 824 bp insert of pVAX14 (Figure 3A) showed two GC-rich regions identified by the European Molecular Biology Open Software Suite (EMBOSS) [16] (nucleotide 774-802 and nucleotide 990-1018) and a consensus human specific CpG (nucleotide 1213-1219). In the novel inserted sequence, 6 open reading frames (ORFs) were identified, depicted by start codons at positions 787, 792, 896, 1207, 1406 and 1495 using the CLC Sequence Viewer (Aarhus, Denmark) software (Figure 3A, Supplementary Figure S1A, Supplementary Table S3). Apart from the inserted sequences, no mutations were detected in the pVAX1 backbone (Supplementary Figure S1A). To demonstrate the expression of these ORFs, the pVAX14 plasmid was transfected in sub-confluent monkey (COS) or human (Phoenix) derived cells and the mRNA transcripts of the ORFs detected by RQ-PCR using the primers shown in the Supplement (Supplementary Table S2). Transcription of five of these ORFs was obtained (ORFs 1, 2, 3, 5 and 6) (Figure 3B), ORF 4 was too small (29 bp) to allow mRNA detection. Although 6 predicted peptides sequences could be deduced from the inserted sequence, none showed homology to a known protein in Genbank databases (Supplementary Table S3).

The potential immunogenicity of the 10 peptides synthesized to cover the 6 ORFs was tested in normal FVB/N mice. Mice received peptide injections as illustrated in the protocol (Figure 3C) and sera were harvested on day 35 (D35) and assayed for Ig production. Only 4 of the 10 peptides induced a significant increase of Ig (Figure 3C); these were peptides 1, 2, 5-1 and 5-2 (the latter two are overlapping peptides of ORF 5). Of note, no significant signs of adverse effects were observed after peptide injections or in the pVAX14-treated mice as compared to controls (Supplementary Figure S1B). In normal FVB/N mice, apart from PB memory T-cells (memT) as expected of DNA (Supplementary Figure S1C), injections of pVAX14 did not differ significantly from the placebo treatment: i.e. no significant differences in interferon gamma (IFNγ) producing cells (Supplementary Figure S1D) and the adapter *MyD88* expression (Supplementary Figure S1E).

### PVAX14 vaccination induced antibody responses in an APL preclinical model

**Figure 4:**
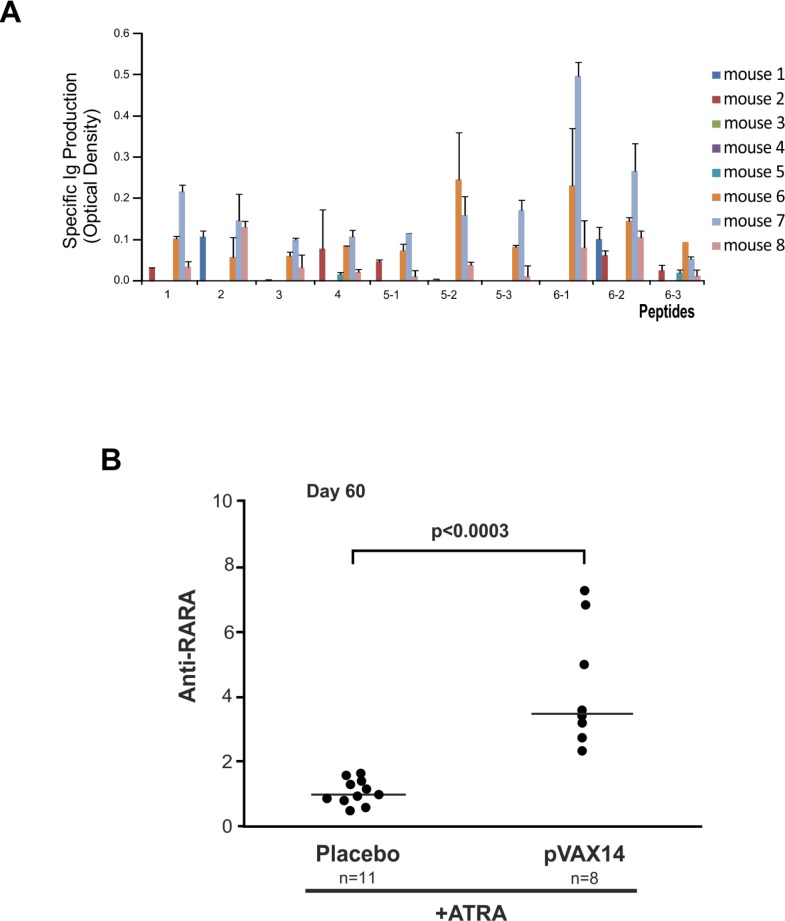
Antibody production of pVAX14-treated APL mice **A.** pVAX14 induced antibody production to the predicted peptides. The absorbance obtained from the sera assayed on day 60 of each mouse treated with ATRA (5mg) + pVAX14 (*n* = 8) was normalized to values obtained from sera from mice treated with ATRA (5mg) alone (*n* = 3) by subtracting the ATRA alone values from those of the ATRA+pVAX14 treated group. Three of 8 mice formed antibodies to all of the peptides. Data are presented as mean +/− SEM of 3 assays; **B.** Anti-RARA antibody production in ATRA+pVAX14 treated APL mice compared to ATRA+Placebo treated mice. The Y-axis represents anti-RARA production expressed as follows: the ratio of specific absorbance (SA)/anti-RARA antibody 9alpha was calculated and this ratio was then divided by the median ratio obtained in control mice (ATRA alone). Increased anti-RARA antibody production in ATRA+pVAX14 treated APL mice (*n* = 8) compared to ATRA+Placebo treated mice (*n* = 11) on day 60 (*p* < 0.0003); nonparametric, unpaired, two tailed, Mann-Whitney test was used to compare different groups. The Prism software was used for the Mann-Whitney test analysis.

When used as a vaccine in combination with ATRA in the APL model antibodies to all of the predicted peptides were observed in the sera of some of the mice (Figure 4A). Three out of 8 treated mice (mouse nos. 6, 7, 8) had detectable antibodies to all 10 peptides, 3 had some antibodies to certain peptides (mouse nos. 1, 2, 3) and 2 mice failed (mouse nos. 4, 5) to mount an antibody response.

As we have previously reported with the specific vaccine [8, 9] the combination of ATRA+pVAX14 significantly increased the production of anti-RARA antibodies compared with ATRA alone. To identify the impact of pVAX14 on the production of anti-RARA antibodies sera from mice treated with ATRA alone or combined to pVAX14 were tested by ELISA (Figure 4). Our results show that like the specific vaccine ATRA+pVAX14 significantly increases the production of anti-RARA antibodies (Figure 4B, *p* < 0.0003).

### Biomarkers of pVAX14 efficacy

In order to determine markers of clinical outcome of pVAX14 treatment, we have compared the level of IFNγ producing cells, memT and *MyD88* expression in placebo- and pVAX14-treated APL and HR-MDS mice. We have also evaluated the cytotoxic effects of CD3+ cells originated from pVAX14-vaccinated animals against APL and HR-MDS BM precursors.

**Figure 5:**
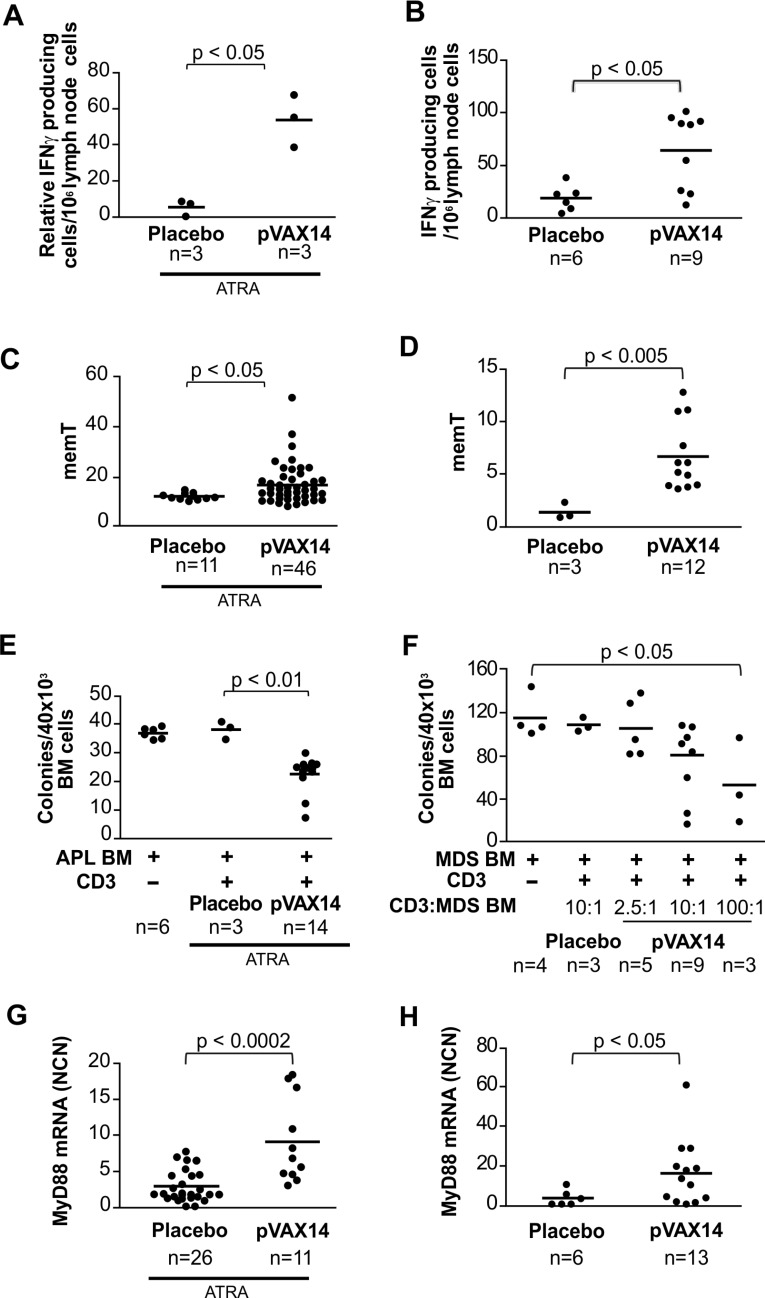
Biomarkers of pVAX14 efficacy **A.** The determination of IFNγ producing cells in the lymph nodes of APL treated mice after stimulation with APL cells was assessed by ELISPOT; mice treated with ATRA or ATRA+pVAX14 showed an increase in IFNγ producing cells in the DNA treated group on day 60 (one-tailed Mann Whitney test); **B.** the determination of IFNγ producing cells in the lymph nodes of HR-MDS treated mice was assessed by ELISPOT; pVAX14 significantly increased IFNγ producing cells in high risk MDS mice on day 30; **C.** memory T cells (memT cells), percentage of CD44^hi^/CD62L^lo^ population within the CD4+ as a measure of memT cells was undertaken on PB of the mice treated with the ATRA+placebo or ATRA+pVAX14. Significantly increased memT cells were observed in the pVAX14 treated APL mice on day 40; **D.** pVAX14 significantly increased memT cells of HR-MDS mice on day 30 (one-tailed Mann Whitney test); **E.** inhibition of APL CFU by CD3+ cells of APL treated mice; fresh BM APL cells from an APL mouse with a high leukemic blast count were used as targets of CD3+ enriched spleen cells of treated APL mice on day 60 in a methycellulose cell culture assay. The ratio of BM to CD3+ was 1:10. CFU colonies were counted at day 7 of incubation. CD3+ cells from ATRA+pVAX14 treated mice significantly reduced APL CFU compared to CD3+ cells from ATRA only treated mice (p < 0.01); **F.** inhibition of HR-MDS CFU by CD3+ cells of HR-MDS treated mice. BM cells from the mice of high leukemic burden were used as targets of CD3+ enriched cells isolated from the spleen of MDS mice on day > 60 of the protocol after the third DNA injection; cells enriched for CD3+ effectors (E) from placebo (HBSS) or pVAX14 treated mice cultured with MDS BM targets (T) in methocult at an E:T ratio of 2.5:1, 10:1 or 100:1 and compared with the MDS BM target alone. Colonies were counted at day 7; the difference between the E:T 100:1 and MDS BM target was significant (*p* < 0.05); **G.** Detection of TLR activation by increased *MyD8*8 expression in pVAX14 treated APL mice. Normalized copy number (NCN) on day 40 of protocol showed that APL mice treated with ATRA+pVAX14 had significantly higher levels of *MyD88* expression compared to APL mice treated with ATRA+placebo; **H.** significantly increased MyD88 expression of HR-MDS mice treated with pVAX14 assayed on day 50 after treatment. Unless stated nonparametric, unpaired, two tailed, Mann-Whitney test was used to compare different groups using the Prism software.

We have previously shown [7, 8] an increase in IFNγ production in APL mice treated with the ATRA and specific vaccine after stimulation with either irradiated APL cells or PMLRARA specific peptides. As shown in Figure 5A, pVAX14 in combination with ATRA also increased the numbers of IFNγ producing cells in both APL stimulated by APL cells (Figure 5A) or specific PML-RARA peptides (data not shown). Treatment with pVAX14 significantly increased the number of IFNγ producing cells in the HR-MDS preclinical model (Figure 5B; *p* < 0.05).

Percentages of memT were significantly higher in ATRA+pVAX14-treated APL (Figure 5C; *p* < 0.05) as well as HR-MDS (Figure 5D; *p* < 0.01) mice compared with control (ATRA in APL and placebo in HR-MDS) mice. The memT levels of pVAX14 and *PML-RARA*-treated groups were similar (Supplementary Figure S2A).

To evaluate the cytotoxic activity of T cells against precursor cells, we applied assays described by Quintarelli et al [17] of APL or HR-MDS BM targets and CD3+ spleen cells from ATRA- or ATRA+pVAX14-treated APL and placebo- or pVAX14-treated HR-MDS mice, respectively. CD3+ cells co cultured with diseased BM were assessed for colony-forming capacity. As shown in Figure 5, CD3+ cells from ATRA+pVAX14-treated APL (Figure 5E) and pVAX14-treated HR-MDS (Figure 5F) had specific cytotoxic effects against APL and HR-MDS precursors, which resulted in a consistent and significant reduction of CFU compared to the culture of CD3+ cells from ATRA-treated APL or placebo-treated HR-MDS mice. These APL-derived effectors had no effect on normal FVB/N BM CFUs (Supplementary Figure S2B). Furthermore depletion of CD4+ cells of APL long term survivors showed that these cells were required to maintain remissions (Supplementary Figure S2C).

Transcript levels of *MyD88*, an adaptor molecule downstream of toll-like receptor (TLR)s signalling were significantly higher in APL mice (Figure 5G) treated with pVAX14+ATRA compared to mice treated with ATRA alone (*p* < 0.0002) as well as in the pVAX14-treated group of HR-MDS mice (Figure 5H) compared to the placebo-treated control (*p* < 0.05).

Taken together, in two different preclinical models of myeloid malignancies (APL and HR-MDS) the survival advantage induced by the pVAX14 vaccine was concomitant with increases in IFNγ producing cells, memT, leukemic precursor-specific CD3+ cells and *MyD88* expression.

## DISCUSSION

As we had previously reported with the specific *PML-RARA* in the APL model [8], the non-specific pVAX14 in the APL and also in HR-MDS preclinical models was shown to be effective in controlling disease and prolonging survival. Whether for the *PML-RARA* specific DNA vaccine in the APL model [7, 8, 14, 9] or the pVAX14 non-specific DNA vaccine in both models, the responses obtained were those known for DNA-based immunotherapy. These include activation of T-cell mediated responses measured by the inhibition of leukemic progenitor growth accompanied by increased IFNγ production, memT, upregulation of *MyD88* and in the case of the transplantable APL model, relapse of long-lived mice after CD4+-cell depletion. We had previously shown with the specific vaccine *PML-RARA* the benefit of its combination with ATRA, not only on improving survival but also in enhancing biomarkers of response [7].

In the APL mouse, prolonged survival was correlated with *PML-RARA* MRD reduction and reduction of BM blasts. As this is a transplantable model long term survivors were obtained and some of these were followed up to 2 years, close to the lifespan of a mouse. For the HR-MDS transgenic mouse model treated with pVAX14 alone, we took advantage of novel biomarkers of the disease we have efficiently used in another novel therapeutic approach [18], and showed that RAS signaling protein dephosphorylation of MEK detected by NanoPro was restored. Despite the persistence of disease as detected by no change overall of the PB Mac1^+^/Gr-1^lo^ population, treatment with pVAX14 reduced the BM blasts enabling the mice to cope with their disease and prolonged life-span. As this is a transgenic mouse model the mice inevitably relapsed once the protective effects were lost and the cells expressing the transgenes expanded.

Vaccination strategies employed in anti-leukemia immunotherapy to date have fallen largely in two categories. Firstly, vaccines against known antigens or epitopes which are peptide-based, such as PR1, a 9-mer peptide of proteinase-3 with high predicted binding and Wilm's Tumor (WT)-1 are shown to lead to specific T-cell responses in myeloid leukemia patients [19]. Secondly, vaccines using primarily whole cells such as leukemic dendritic cells, cell lysates and tumor cell lines modified to produce cytokines have been reported in AML clinical trials [20, 21, 22, 23]. For example, administration of autologous dendritic cells (DC) loaded via electroporation with *WT-1* mRNA resulted in complete remission in 50% of AML patients in a phase I/II study [24].

However, targeted vaccine approaches have limitations. Peptide-induced immune responses towards cells expressing only one epitope do not induce memory of the anti-tumor immune responses, explaining why the effects are short-lived. Likewise, cell based therapies such as modified dendritic cells are specific to each individual [25, 26]. DNA vaccines are not restricted to a given epitope or to a given individual. DNA vaccines have the disadvantage of being weak inducers of antitumor effects on their own (reviewed in [27]), and require being associated with agents that enhance immune response and/or delivery. Granulocyte macrophage-colony stimulating factor (GM-CSF) has often been added to vaccines or used as a vaccine itself [28, 29, 30] and electroporation with the DNA injection has been effectively used to boost immune responses [1]. The DNA encoding an immunogenic peptide is taken up by either the muscle cells or antigen presenting cells (APCs); so this is analogous to an *in vivo* transfection [3]. The muscle cell then processes the sequences and antigen is released, seen by B and T-cells to induce both humoral and cytotoxic T-cell responses [3]. That we observe antibody responses to each of the peptides following pVAX14+ATRA treatment of APL cells supports the production of a humoral response following pVAX14 DNA injections. Clinical trials with DNA plasmids have shown enhancement of specific immune responses including antibody production, proliferation of CD4+ and CD8+ T lymphocytes, antigen-dependent cytotoxicity, and cytokine secretion [31]. Many DNA plasmids harbour CpG motifs in their sequence, which signal through TLR9, leading to the induction of antigen presentation through major histocompatibility complex (MHC)-I, MHC-II molecules, co-stimulatory molecules expression and cytokine secretion [31]. CpG containing oligonucleotides have been used in clinical trials [32, 33], but the ease of manufacture and stability of plasmids make them attractive tools with increased signalling towards NF-κB as observed with upregulation of *MyD88* upon treatment with pVAX14. Furthermore, our construct contains 5 ORFs, which we show to be transcribed and encoding immunogenic peptides, to which vaccinated APL animals develop antibodies.

In our previous studies, we had highlighted the documented effects of Vitamin A and ATRA on the immune system such as increased proliferation of T lymphocytes [34, 35], increased synthesis of Igs by B lymphocytes [36], and the induction of differentiation of dendritic cells to enhance antigen presentation [37]. More recently, ATRA has been shown to suppress myeloid derived suppressor cells [38]. We showed that a specific vaccine in combination with ATRA rescued 50% of the APL mice from relapse and death unlike DNA or ATRA alone where all the mice relapsed and died; this protective effect was T and B-cell mediated [7, 8, 9]. This stresses the importance of ATRA as an immunomodulator.

Tumor associated antigens have not yet been identified for all tumors and in hematological disorders multiple factors may be involved and major oncogenic proteins have not yet been identified impeding the transfer of the concept. Non-specific boosting of the anti-tumor response has been used in clinical practice as early as *bacillus* Calmette-Guerin (BCG); a BCG based vaccine has been shown to protect against cancers [39]. In AML, the combination of histamine and interleukin-2 (IL-2) has been shown to stimulate immune responses and achieve prolonged survival in a Phase III study, however, side effects were limiting [40]. The pVAX14 DNA approach appears thus a potential alternative approach. DNA vaccines are well tolerated and offer a good alternative to control progression to leukemia and to prevent relapse [41].

Although past history has shown the good tolerance of DNA vaccines, regulations are in place to ensure that vaccine manufacture provides a safe product. Preclinical studies have shown that DNA vaccines are unlikely to cause systemic auto-immune disease, and that at the administered doses, integration has so far not been an issue. Preliminary toxicology test of pVAX14 in rodents suggest absence of toxicity (data not shown). PVAX14 comprises the pVAX1 backbone, which has no mutations and this vector is already used in clinical practice with no reported adverse effects [42].

We hypothesize that the pVAX14 construct is acting as an adjuvant, boosting pre-existing immune responses initiated by the antigens from the malignant cells as evidenced by the increase in anti-RARA antibodies in the APL model and consequent reduction in *PML-RARA* transcripts. It has been shown that chemotherapy such as anthracyclins can induce immunogenic cell death ([43] and reviewed in [44]) and azacitidine, the standard treatment for HR-MDS patients is also an immunomodulator [45]. Extended survival obtained in the HR-MDS model together with dephosphorylation of the RAS signalling protein MEK shows its effect in this mouse model. Only a phase I trial in humans will provide the required data of safety and efficacy; however, our data confirm progress in the field of anti-tumor immunotherapy and underscore the potential of a non-specific DNA vaccine to control myeloid diseases in preclinical models. The efficacy of a vaccine is determined by the magnitude, duration and quality of the immune response it induces. Depending upon the disease, both the innate and adaptive arms of the immune system may contribute to protection. Despite the biological heterogeneity of different cancers, which may influence the immune system in different ways, adjuvants such as DNA, may have efficacy in a variety of cancers where immunogenic apoptosis has been induced by either the pro-apoptotic features of the disease or by previous therapy or in combination with an immunomodulator.

## MATERIALS AND METHODS

### Plasmids (pcDNA3PML-RARA, pVAX1 and pVAX14)

The specific *pcDNA_3_PMLRARA-FrC* (*PML-RARA*) construct previously reported by us [7, 8, 9] included the pcDNA3 vector, which is now limited on clinical use due to its ampicillin resistance. PVAX1 is a plasmid vector designed to be consistent with the Food and Drug Administration (FDA) requirements (Docket no. 96N-0400, 1996) and accepted for clinical trials as based on kanamycin resistance for selection. We thus engineered a construct, consisting of the DNA plasmid vector pVAX1, with an inserted novel nucleotide sequence of 824 bp, which arose as a recombination between part of the kanamycin resistance gene (nucleotides1226-1701) and the pVAX1 adjacent sequence (nucleotides 873-1225) inverted and reversed so that the antisense strand became the sense strand in reverse, hence we named this construct pVAX14Flipper (pVAX14). The insert recombined into the BamHI site of the multiple cloning site fused in frame to the pVAX1 backbone to create an open reading frame (ORF) and abolishing the bovine growth hormone polyadenylation site (BGH poly A). The clone was sequenced using the Genomic and Sequencing Platform of Institut Cochin (Paris) and the insertion was stable.

### Expression of open reading frames (ORFs)

Plasmid DNA (20 mg or 15 mg) was introduced into monkey kidney cells (COS) or human Phoenix cells via electroporation or calcium phosphate as per manufacturer's instructions (Sigma). After 48 h incubation at 37° C under 95% air/5% CO_2_, cells were harvested and RNA extracted immediately. Gene expression was calculated for each probe set (Table S2) using the comparative Delta Delta Ct (ΔΔCt) method [46] of empty vector or pVAX14-vector against the untransfected RNA, all normalized against *ABL* as the house keeping gene.

### Animal models and treatment protocols

BM and spleen cells were prepared as previously described [8]. All mice were of the Friend leukemia Virus B strain from the National Institutes of Health (NIH) (FVB/N) and were maintained under pathogen free conditions in the barrier facilities of the Institut Universitaire d'Hématologie. Procedures involving animals and their care conformed to institutional guidelines that comply with national and international laws and policies and were authorized by the local ethical committee (Committee on the Ethics of Animal Experiments - Paris Nord C2EA-121, approval no. 2014-IUH006).

### Tissue and cell preparation

Differential blood counts were obtained using an automated hematology analyzer (Cell Dyn, Abbott Diagnostics, France or MedoniCA620, Stockholm, Sweden). White PB cells were analyzed by flow cytometry as previously described [15] (FACS Calibur, Becton Dickinson, San Jose, California, USA). BM was obtained by flushing long bones with RPMI media (L-Glutamine 200 mM, 2% Fetal Bovine Serum, penicillin/streptomycin). PB and BM smears were prepared according to standard hematological techniques. The cells were washed with phosphate buffered saline (PBS) at 400 g for 10 min at 4°C. The pellet was resuspended in 10 ml of PBS. Cell counting was performed in the presence of Nigrosine or trypan blue (0.4%), which is taken up by dead cells. Cells were centrifuged and resuspended in sterile PBS for transplants injected intravenously (IV). Murine spleen cells were harvested and disrupted in RPMI.

### APL model

A transplantable APL model derived from transgenic mice with the PML-RARA breakpoint cluster region 1 (bcr1) of APL was used and treated with ATRA (5mg, Innovative Research of America, Sarasota, Florida, USA) as previously described [13]. Sampling was done on days indicated in the Figure legends. Disease was monitored by clinical and blood measurements and by *PML-RARA* mRNA for minimal residual disease (MRD). As we had observed previously that depletion of CD4+ cells resulted in relapses, CD4+ cells were depleted in long term survivors (120 days) by injecting anti-CD4 antibodies weekly as we previously described [7].

### HR-MDS model

Mutant neuroblastoma-Rasheed sarcoma virus cellular homologue *(NRAS)D12* with a substitution of aspartic acid at amino acid position 12 and B cell lymphoma 2 (*BCL-2*)-mediated HR-MDS mice as we previously described were used [15]. Briefly, *NRASD12* mice were crossed with hemizygote mouse mammary tumor virus long terminal repeat (*MMTV)* transactivator *(tTA)* mice *(MMTVtTA)* mice. Tetracycline inducible *BCL-2* mice (*TetoBCL-2.1* line) [15] were crossed with hemizygote *MMTVtTA* mice. The double transgenic *MMTVtTA/NRASD12* mice were then crossed with the *MMTVtTA/TBCL-2* mice to generate *MMTVtTA/TBCL-2/NRASD12* mice. The disease is described as similar to HR-MDS in patients [15]. The mice were bred and genotyped using standard husbandry and PCR techniques as described previously [15]. PB BCL-2 expression was performed by flow cytometry using the human specific fluorescence isothiocyanate (FITC) conjugated anti-BCL-2 antibody using manufacturer's protocols (BD Pharmingen, San Diego, CA, USA). PB was labelled with the homing cell adhesion molecule (Mac-1) conjugated to phycoerythrin (PE) and GPI-linked myeloid differentiation marker (Gr-1) conjugated to FITC obtained and used according to the Manufacturer's protocols (BD Pharmingen, San Diego, CA, USA). All measurements were performed using BD FACS Calibur or Facs Canto flow cytometer. Analysis was performed using the Cellquest software. Mice were followed for disease by measuring blood counts (Medonic CA620, Stockholm, Sweden) and PB BCL-2 and increased Mac1^+^/Gr1^lo^ levels, within which the blast cell population is located and recruited to trial once the platelet counts fell below 1300 × 10^3^/mm^3^ at 3-6 months of age. This reduction in platelet count and their recovery upon treatment tracks the disease well and correlates with survival [15]. This disease is transplantable and was assessed by IV injection of spleen cells (10^7^) of diseased mice into lethally irradiated (5 Grays twice with an interval of 4 hours) FVB/N syngeneic recipients [7, 15, 8, 14].

### Vaccination protocol

For normal (FVB/N), APL and HR-MDS mice, 2×50μg DNA in the vehicle [Hepes buffered saline solution (HBSS)] was injected intramuscularly (IM) in each of the quadriceps every 20 days for 3 cycles. Blood samples were collected at different time points to monitor disease and biomarkers. Control mice were treated with IM injections of the vehicle.

For APL 10^4^ cells were transplanted by IV injection into syngeneic 6-8 week old FVB/N mice. During the course of the study, two lines derived from transgenic mice (935 and 909), which gave identical APL-like diseases, were used. Treatment of the APL model: ATRA 21-day slow release pellets (5 mg) (Innovative Research of America, Sarasota, Florida, USA) were administered on day 6 after injection of APL cells. Control APL mice were treated with IM injections of HBSS [8]. Sampling was done on days indicated in the legends. Mice were monitored by clinical and blood measurements to monitor disease, as previously and *PML-RARA* mRNA for MRD detection.

### Real time quantitative polymerase chain reaction (RQ-PCR)

The RQ-PCR method using Power SYBR Green for ORFs, *PML-RARA* and *MyD88* (Applied Biosystems) and primer sequences used are detailed in Supplementary Table S2. The human *PML-RARA* clone [47] and the mouse *MyD88* cloned by our laboratory were used to generate the standard curves. The reference gene used was mouse *Abl*, which was cloned by our laboratory. Target genes were ran for each sample in triplicate. In order to quantify the samples, a standard curve was software generated according to the Ct obtained with the diluted plasmids. The copy number of the gene of interest for each sample was determined as a function of the detected fluorescence extrapolated from the standard curve. The pVAX14+ATRA-treated group was stratified according to survival below or above the median of individual protocols.

### Isoelectric focusing protein analyses (NanoPro)

Splenocytes (1×10^6^) were lysed in radio-immunoprecipitation assay (RIPA) buffer and subjected to a nanofluidic proteomic immunoassay (NIA) run on the NanoPro1000 [48] (ProteinSimple, Santa Clara, CA) using mitogen-activated protein/extracellular signal-regulated kinase kinase (MEK) antibodies. Proteins were separated according to their isoelectric point (ip). Protein (0.1 mg/ml of lysate) in a final volume of 15μl was loaded in a 384-well plate with 5-8 gradient ampholyte mix. Antibodies were diluted in ProteinSimple antibody diluent: mitogen-activated protein kinase (MEK1 (Upstate 07-641, Millipore, Billerica, MA, USA). Actin (BD Bioscience, Paris, France) was used as a control for protein loading. Samples were run in triplicate.

### Immunogenicity of ORF predicted peptides sequences

Peptides encoded by the sequences delineated (ORFs 1-6, Supplementary Figure S1A) were synthesized as shown (Supplementary Table S3) and tested for immunogenicity in normal FBV/N mice. As ORFs 5 and 6 were 60-mers, 3 overlapping peptides for each were synthesized. Briefly, plates coated with each of the peptides were cultured with sera from peptide treated mice and immunoglobulin (Ig) G production was measured.

### Evaluation of biomarkers

Anti-RARA or anti-pVAX14 peptide antibody levels were measured by ELISA using a method described previously [8].

Briefly, for RARA antibody detection sera from vaccinated APL mice were added to plates coated with recombinant RARA protein. Each test was performed in duplicate. Specific absorbance (SA) was calculated by the difference between the absorbance measured in the wells coated with GST-RARA and the wells coated with control GST. To minimize inter-assay variations, the specific absorbance of each serum was divided by the specific absorbance (SA) of 9α MAb (RAR - 9A6 Euromedex, Mundolsheim) diluted 1/5000, to give the ratio SA/9α and this ratio was then divided by the median ratio obtained in control mice (ATRA alone).

For each synthetic peptide, 3 FVB/N mice were immunized by one subcutaneous injection with 100μg of peptide in 100 μl V/V phosphate buffered saline (PBS)/Complete Freund's Adjuvant (CFA), and three injections intraperitoneal one week, three weeks and 30 days later with the same peptide without CFA (see protocol illustrated on Figure 1C). Sera were collected before immunization and four days after the last injection and were tested by ELISA. 96-well ELISA microplates were coated with diluted peptides (500 ng per wells) for 2hrs at 37°C (dilution was performed in 50mM carbonate/bicarbonate buffer, pH 9.5). For the antibodies generated by pVAX14+ATRA, day 60 sera from mice (protocol illustrated on Figure 1A) were used. After coating the microplates were washed five times in PBS/0.05% Tween 20 and incubated 1h at room temperature with 50 μl per well of PBS containing bovine serum albumin (BSA). Microplates were washed five times (washes and dilution were performed in PBS with 0.05% Tween 20) and incubated 90 min at 37°C with 50 μl serum 1:50 diluted (each serum in triplicate). After washing, the microplates were incubated 90 min with 50 μl per well of peroxidase-conjugated goat anti mouse immunoglobulin (Jackson) diluted 1:20,000. The microplates were washed five times and peroxidase activity was revealed by adding 100 μl per well of either Sigmafast OPD for the sera generated with peptides (optical density (OD) as a measure of Ig production was measured using a Dynatech, MR5000 (Labsystem, Cergy, France) at a wavelength of 495 nm) or tetramethylbenzidine (TMB) (BD Bioscience^®^) for the sera generated by pVAX14+ATRA (OD was measured using a Dynatech, MR5000 at a wavelength of 450 nm versus 550 nm). The reaction was stopped 3 to 20 min by addition of 25μl of 2.5N H_2_SO_4_ per well. The pVAX14+ATRA values were normalized to the ATRA values by subtracting the mean of 3 ATRA treated mice assayed in triplicate from the mean of each sample of pVAX14+ATRA values assayed in triplicate. For each of the 10 peptides covering the 6 ORFs of pVAX14 SA was measured.

### Interferon gamma (IFNγ) production

IFNγ producing cells were measured using an Elispot kit according to the manufacuters' instructions (Becton Dickinson (BD, Berges, France). Briefly, lymph nodes were harvested at different time points after the DNA injections as indicated in Figure legends. Lymph node cells (LN, 10^6^) were incubated overnight in wells coated with anti-IFNγ-antibody. Biotinylated anti-mouse IFNγ (2 μg/ml) was used to detect the captured IFNγ. Spots were visualized using Streptavidin-Horse Radish Peroxidase (SVa-HRP) and 3-amino-9-ethylcarbazole (AEC) substrate (Becton Dickinson) followed by image analysis. Irradiated (25 Gy) APL spleen cells (0.25×10^6^ per 10^6^ LN) or PML-RARA peptide (2 μmolar per 10^6^ LN) [ASGAGEAAIETQSSS (15-mer)] were used to stimulate effectors from treated APL mice as spontaneous release was similar to healthy mice. Relative numbers of IFNγ producing cells were calculated by subtracting the unstimulated values from the stimulated numbers. Spontaneous release of HR-MDS lymph node cells were assayed without the need for stimulating as in a transgenic model the transgenes continue to be expressed and the lymph nodes were stimulated *in vivo*.

### Measurement of memory T-cells (memT) by flow cytometry

The determination of percentage of the homing cell adhesion molecule (HCAM) or CD44^hi^/CD62L^lo^ or L-selectin population within the CD4+ as a measure of memT was undertaken on peripheral blood (PB) of the mice. The antibodies were obtained from BD Biosciences (Paris, France). Samples were measured using a BD FACS Canto flow cytometer and at least 10,000 events were collected from each sample. Data were processed using the BD FACS Canto II Software.

### CFU assay

A modified method previously described [17, 49] to assess the effects of CD3+ effectors on BM progenitors were applied. Briefly, BM cells from diseased mice with high percentage of blasts were used as targets. CD3+ enriched cells from the spleen of treated mice using CD3 magnetic beads from Miltenyi Biotec (Bergisch Gladbach, Germany) were co-cultured with BM from normal FVB/N or diseased mice. Colonies from APL BM were picked and PML-RARA bearing cells were confirmed by PCR (data not shown). The ratio of CD3+ effector cells (E) to BM target (T) for culture in Methocult^®^ media as recommended by the manufacturer (Stem Cell Technologies, Vancouver, Canada). This assay kit contained rm-Stem Cell Factor, rmIL-3, rhIL-6 growth factors and insulin and transferrin. Briefly 10^6^ BM cells were centrifuged and resuspended in 0.5 ml Iscove's media supplemented with 2% heat inactivated fetal calf serum (FCS), 2 mM glutamine, 5 UI/ml penicillin and 300 mg/ml streptomycin. 0.3 ml of cells was added to 3 ml of Methocult^®^ and 1 ml (2×10^4^) was plated per 35 mm dish (in triplicate). Cultures of BM from diseased mice and enriched CD3+ spleen cells from treated mice were plated at an effectors to target (E:T) ratio of 2.5:1, 5:1 and 10:1 and incubated for 7 days at 37° C, 5% CO_2_ in air and > 95% humidity. Identification and counts of colonies on day 7 were done according to the technical manual of the manufacturer. The optimal ratio (E:T) for APL was 10:1. For MDS an additional ratio of E:T of 100:1 was used.

### Survival and statistical analysis

Kaplan-Meier survival curves were used to analyse survival of the various groups of APL mice treated with placebo (HBSS), pVAX14 alone, ATRA alone, ATRA+pVAX14 or ATRA+PML-RARA and of HR-MDS mice treated with placebo or pVAX14. Comparisons of survival employed the Mantel-Cox (log-rank) test. Unless otherwise stated, other comparisons employed the Student's t-test, except where *n* < 10 when the Mann-Whitney test was used.

## SUPPLEMENTARY MATERIAL FIGURES AND TABLES


