# Supplementary material for: DNA-mediated adjuvant immunotherapy extends survival in two different mouse models of myeloid malignancies
Source: Oncotarget. 2015 Sep 10;6(32):32494–508. doi: 10.18632/oncotarget.5572 (PMC4741708; doi:10.18632/oncotarget.5572)
Supplement: Supplementary file 1 [file oncotarget-06-32494-s001.pdf]

DNA-mediated adjuvant immunotherapy extends survival in two different mouse models of myeloid malignancies

Supplementary Material

Table S1. Statistical analysis of APL survival curve Figure 1B
Log-rank (Mantel-Cox) test was used to compare the percent survival of different groups. Prism software was used for the Mantel-Cox (log-rank) analysis

	log-rank (Mantel-Cox)	
	Chi-2 value	p value
ATRA+pVAX14 vs Placebo	70.96	P<0.0001
ATRA+pVAX14 vs ATRA	10.14	P<0.0014
ATRA+pVAX14 vs PVAX14	58.51	P<0.0001
ATRA+pVAX14 vs ATRA+PML-RARA	0.56	P<0.46
ATRA+PML-RARA vs Placebo	55.45	P<0.0001
ATRA+PML-RARA vs ATRA	15.52	P<0.0001
ATRA+PML-RARA vs pVAX14	45.16	P<0.0001
pVAX14 vs Placebo	4.9	P<0.03
pVAX14 vs ATRA	35.88	P<0.0001
ATRA vs Placebo	46.07	P<0.0001

Table S2. Primer sequences for open reading frames (ORFs), *MyD88*, *PML-RARA* and *ABL*

Primer	Sequence (5'->3')	Amplicon bp
ORF1-F	ATGCGATGTTTCGCTTGGTGGTCGA	108
ORF1-R	TCACCTTGCTCCTGCCGAGAAAGTA	
ORF2-F	ATGTTTCGCTTGGTGGTCGAATGGG	108
ORF2-R	TCATCTCACCTTGCTCCTGCCGAGA	
ORF3-F	ATGACAGGAGATCCTGCCCC	63
ORF3-R	TCACTGAAGCGGGAAGGGAC	
ORF5-F	ATGCCTGCTATTGTCTTCCCAATCC	180
ORF5-R	CTATTGTCTTCCCAATCCTCCCCCG	
ORF6-F	ATGCAATTTCCTCATTTTATTAGGA	189
ORF6-R	TTACCAGAGGGCGCCCCAGCTGGCA	
hABL-F	TTCAGCGGCCAGTAGCATCTGACTT	263
hABL-R	GATGTAGTTGCTTGGGACCCA	
mAbl-F	GAAGACCTTGAAGGAGGACACCATG	183
mAbl-R	GGGTACACACCCCTAGCAGCT	
PMLRARA-F	GTCTTCCTGCCCAACAGCAACC	190
PMLRARA-R	CTCACAGGCGCTGACCCCATAGT	
MyD88-F	CGCGCATCGAGGAGGACTGC	156
MyD88-R	CCGGCGTTTGTCCTAGGGGGT	

Table S3. Peptide sequences predicted by open reading frames (ORFs) of pVAX14

1, peptide 1 (ORF 1); 2, peptide 2 (ORF 2); 3, peptide 3 (ORF 3); 4, peptide 4 (ORF 4); 51, peptides 5.2, 5.3 (ORF 5); peptides 6.1, 6.2 6.3 (ORF 6). As the predicted peptides of ORFs 5 and 6 were too large to synthesize 3 overlapping peptides were designed and synthesized for each of these ORFs; overlapping sequences are shown in bold

ORF	Peptide N°	location	Amino Acid Sequence
1	1	787	MRCFAWWSNGQVAGSSVCSRRIASAMMDTFSAGAR
2	2	792	MFRLVVEWAGSRIKRMQPPHCISHDGYFLGRSKVR
3	3	896	MTGDPAPALRPIAASPFPLQ
4	4	1207	MRNDPHPVS
5	5-1	1406	MPAIVFPILPLAVLPH**PTPQNRMTPTQT**
	5-2		**PTPQNRMTPTQT**MRC**NFLILLGKDSGSG**
	5-3		**NFLILLGKDSGSG**TFQGQGRHGGGLGRQ
6	6-1	1495	MQFPHFIRKGQWEWHLP**GSRKARGRIGK**
	6-2		**GSRKARGRIGK**TIAGML**GMRWALWLLLG**
	6-3		**GMRWALWLLLG**GFMDSKRTGIASWGALW

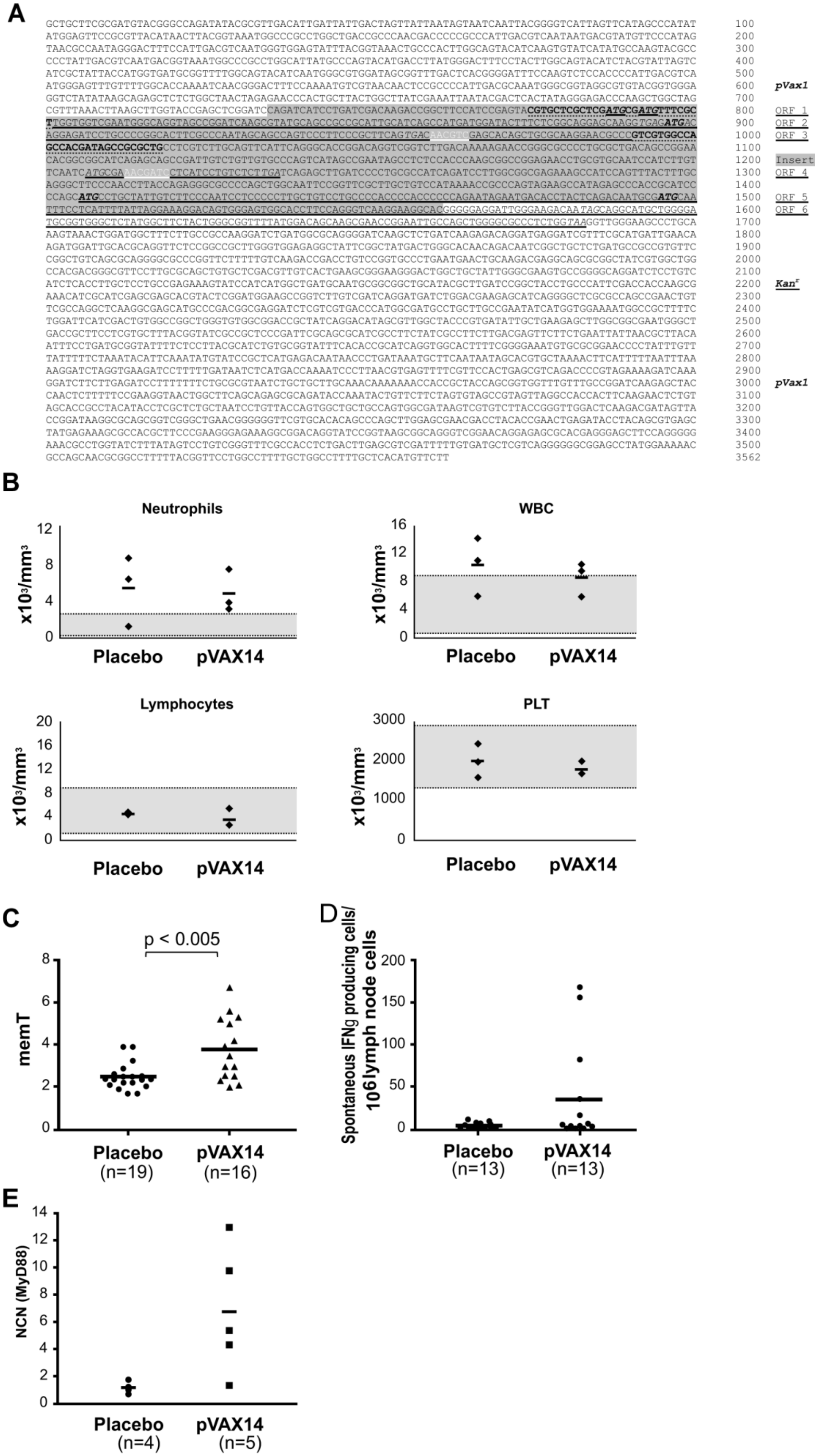
A
100
200
300
400
500
600
pVax1
700
800
ORF 1
900
ORF 2
1000
ORF 3
1100
1200
Insert
1300
ORF 4
1400
1500
ORF 5
1600
ORF 6
1700
1800
1900
2000
2100
2200
Kan^r
2300
2400
2500
2600
2700
2800
2900
3000
pVax1
3100
3200
3300
3400
3500
3562
B
Neutrophils
WBC
Lymphocytes
PLT
x10³/mm³
Placebo
pVAX14
C
p < 0.005
memT
Placebo (n=19)
pVAX14 (n=16)
D
Spontaneous IFNg producing cells/ 10⁶lymph node cells
Placebo (n=13)
pVAX14 (n=13)
E
NCN (MyD88)
Placebo (n=4)
pVAX14 (n=5)

Supplementary Figure S1. Characteristics of pVAX14

A) Sequence of pVAX14 plasmid showing functional domains; **g**rey highlighted sequences from the kanamycin (Kan) resistance gene in the antisense orientation and reversed (Kan insert originally in pVAX1 nucleotides 1226-1701)**,** - in italics and underlined in dashed, the GC-rich sequences, - in white, the mouse and human specific consensus CpG sequences, the open reading frames (ORFs) are underlined with the start codons in bold italics, the pVAX1 sequences in the insert were originally pVAX1 nucleotide nos. 873-1225, the BamHI site (GGATC) in the multiple cloning site just precedes the insert. The entire insert of pVAX1 nucleotides 873-1701 was inverted (the antisense strand was reversed and now the coding strand) and inserted between pVAX1 nucleotide position 734 and 998; B) peripheral blood (PB) counts of normal FVB/N mice immunized with control placebo phosphate buffered saline (PBS) or pVAX14 DNA (50 µg) on day 35 of protocol illustrated on Figure 3C. The normal range **i**s delineated in grey; C) PB memory T-cells (memT) (defined as percentage (%) of $CD44^{hi}/CD62L^{lo}$ within the CD4+ population) were measured on day 40. Controls were mice treated with placebo hepes buffered saline (HBSS); significant differences were observed between pVAX14 and the control ($p<0.005$); D) interferon gamma (IFNγ) producing cells in normal mice treated with control placebo (HBSS) and pVAX14. ELISPOT assays were undertaken on day 40 showing no significant difference between pVAX14 and placebo; E) *MyD88* expression of pVAX14 was similar to control placebo (HBSS). Nonparametric, unpaired, two tailed, student t-test was used to compare different groups. Prism software was used for the t-test analysis.

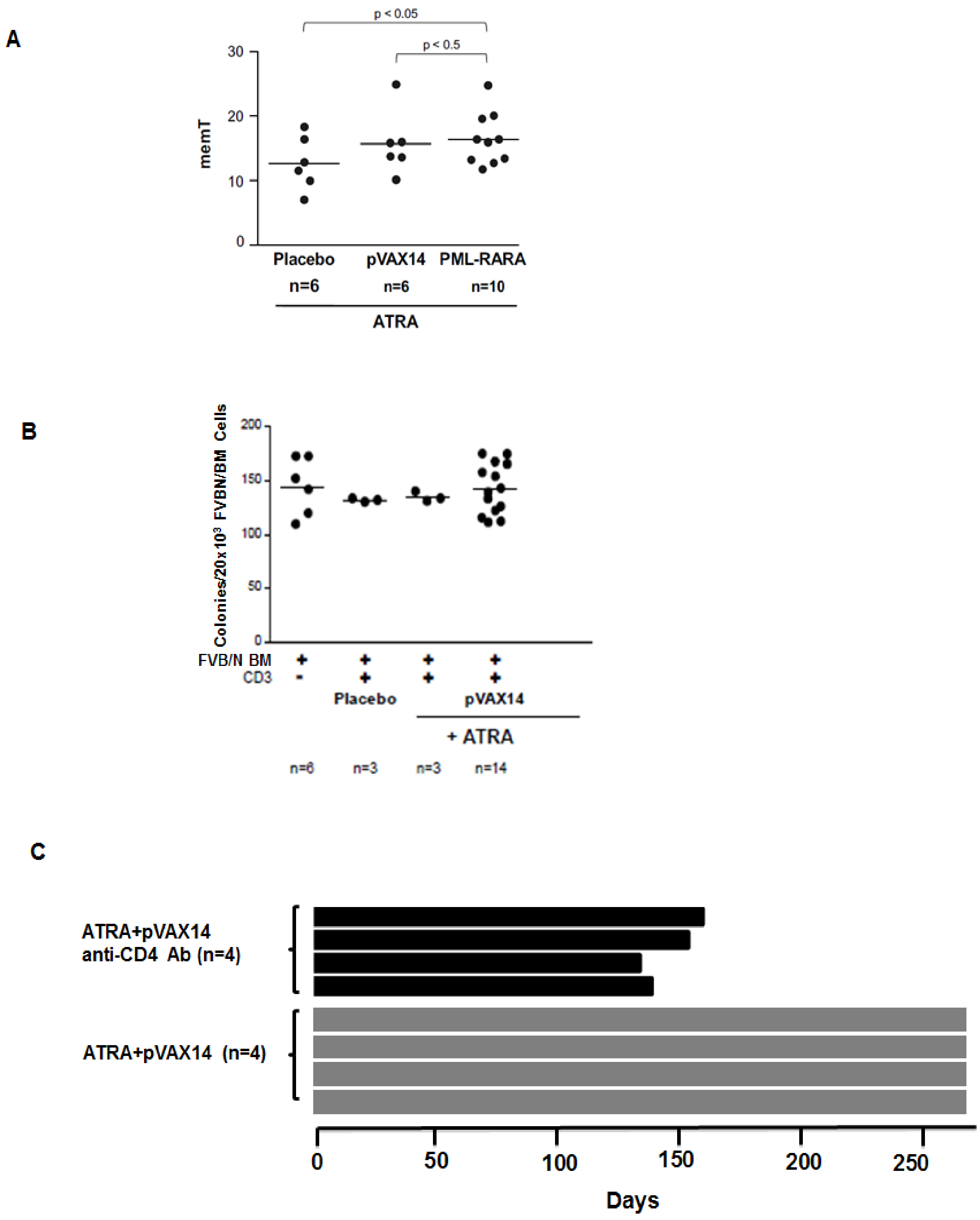

Figure S2 DNA-based immunotherapy induced responses

A) Increase in memory T-cells (memT) of pVAX14 or *PML-RARA* -treated APL mice measured on day 40; **B)** CD3+ effectors from APL mice treated as shown showing no effect on FVB/N progenitors plated at an effector:target (E:T) of 10:1; **C)** The protective effect mediated by ATRA+ pVAX14 in APL mice is CD4+ T-cell dependent. Long term survivors (LTS) from ATRA+pVAX14-treated mice were CD4-depleted by weekly injections of CD4-specific antibody started on day 120 after engraftment of leukemic cells. Control ATRA+pVAX14 treated mice were followed up for >500 days. Nonparametric, unpaired, two tailed, Mann-Whitney test was used to compare different groups. Prism software was used for the t-test analysis.
